# Antimicrobial Activity of Cold Atmospheric Plasma on Bacterial Strains Derived from Patients with Diabetic Foot Ulcers

**DOI:** 10.4014/jmb.2407.07035

**Published:** 2024-09-30

**Authors:** Roopak Murali, Pooja Singh, Divya Ragunathan, Ramya Damarla, Dharshini Kichenaradjou, Kirtanna Malichetty Surriyanarayanan, Satish Kumar Jayaram, Harish C. Chandramoorthy, Ashish Kumar, Mary Elizabeth Gnanambal Krishnan, Rajesh Kumar Gandhirajan

**Affiliations:** 1 Department of Human Genetics, Faculty of Biomedical Sciences and Technology, Sri Ramachandra Institute of Higher Education and Research (SRIHER), Porur, Chennai 600116, India; 2 Department of Biotechnology, Faculty of Biomedical Sciences and Technology, Sri Ramachandra Institute of Higher Education and Research (SRIHER), Porur, Chennai 600116, India; 3 Department of Plastic Surgery, Sri Ramachandra Medical College, Sri Ramachandra Institute of Higher Education and Research (SRIHER), Porur, Chennai 600116, India; 4 Department of Microbiology & Clinical Parasitology, College of Medicine, King Khalid University, Abha 61421, Saudi Arabia

**Keywords:** Diabetic foot ulcer, cold atmospheric plasma, reactive oxygen species, nitric oxide, bacteria

## Abstract

Bacterial infections or their biofilms in diabetic foot ulcer (DFU) are a key cause of drug-resistant wounds and amputations. Cold atmospheric plasma (CAP) is well documented for its antibacterial effect and promoting wound healing. In the current study, we built an argon-based, custom CAP device and investigated its potential in eliminating laboratory and clinical bacterial strains derived from DFU. The CAP device performed as expected with generation of hydroxyl, reactive nitrogen species, and argon species as determined by optical emission spectroscopy. A dose-dependent increase in oxidation reduction potential (ORP) and nitrites in the liquid phase was observed. The CAP treatment eliminated both gram-positive (*Staphylococcus aureus*, *Entrococcus faecalis*) and negative bacteria (*Pseudomonas aeruginosa*, *Proteus mirabilis*) laboratory strains. Clinical samples collected from DFU patients exhibited a significant decrease in both types of bacteria, with gram-positive strains showing higher susceptibility to the CAP treatment in an ex vivo setting. Moreover, exposure to CAP of polymicrobial biofilms from DFU led to a notable disruption in biofilm and an increase in free bacterial DNA. The duration of CAP exposure used in the current study did not induce DNA damage in peripheral blood lymphocytes. These results suggest that CAP could serve as an excellent tool in treating patients with DFUs.

## Introduction

Cold atmospheric plasma (CAP) is a form of ionized gas recognized as the fourth state of matter. This non-thermal plasma is generated at relatively low temperatures and contains various primary and secondary excited atoms, molecules, ions and free radical species. The features of the plasma source significantly impact the chemical composition and the presence of primary and secondary reactive species [1-3]. Numerous research works have illustrated that CAP can effectively neutralize a variety of organisms, such as bacteria, viruses, fungi, yeast, and bacterial spores [4, 5]. CAP is widely applied in the realm of biomedicine, performing tasks such as modifying hydrophilic and chemically active materials, eliminating bacteria, disinfecting skin, managing wounds, treating caries, sterilizing implant surfaces, enhancing blood coagulation, and exhibiting anti-cancer properties [6, 7].

Diabetes mellitus is characterized as a metabolic disorder resulting from compromised carbohydrate, lipid, and protein metabolism stemming from inadequate insulin function. Type 1 diabetes mellitus (T1DM) arises due to an autoimmune response wherein the body specifically attacks the insulin-secreting cells, whereas type 2 diabetes mellitus (T2DM) is caused by an imbalance between the production of insulin by beta cells and its efficacy, resulting in insulin resistance [8]. Prolonged hyperglycemia leads to long-term health consequences and it is estimated that 25% of all diabetes mellitus patients develop chronic wounds, such as diabetic foot ulcer (DFU) [9]. These foot ulcers occur when a particular area of the foot has been subjected to continuous pressure or trauma [10]. The exposed wounds often become infected, further hindering the healing process. If not intervened upon, DFUs have the potential to result in severe complications, such as sepsis, abscess formation, the necessity of amputation, osteomyelitis, and gangrene. The bacterial load observed in the DFU contains gram-positive and gram-negative bacteria [11-13]. The infection caused can be due to a particular bacterial strain (monomicrobial) or by the action of multiple bacterial strains (polymicrobial) [11, 12]. The most commonly found gram-positive bacteria are Methicillin-resistant *Staphylococcus aureus* (MRSA), *Enterococcus* spp., *Staphylococcus aureus*, *Enterococcus faecalis*, *Corynebacterium* spp., and *Staphylococcus epidermidis*. The predominantly found gram-negative bacteria are *Enterobacter cloacae*, *Pseudomonas aeruginosa*, *Escherichia coli*, *Klebsiella pneumoniae*, *Proteus mirabilis*, and *Acinetobacter baumannii*/*haemolyticus* [14]. Among these different bacteria, *Staphylococcus aureus* and *Pseudomonas aeruginosa* were found to be two of the most dominant gram-positive and gram-negative species, respectively, appearing in almost all ulcers. *S. aureus* is known to form biofilms that cause multidrug resistance and prevent host immune response. In addition, *Enterococcus faecalis* is found in most DFUs while *P. aeruginosa* is resistant to antibiotics, resulting in severe consequences like sepsis and amputation [12, 13, 15, 16].

The Indian subcontinent has various ethnic and cultural groups and experiences a wide range of climatic conditions from arid deserts to humid tropical regions, making it exceptionally climatically diverse, while antimicrobial resistance arises from the overuse of antibiotics [17]. Recent studies also indicate antibiotic resistance frequently occurs in natural environments, such as soil reservoirs and regions with increased population densities, while elevated temperatures hasten the acquisition, accumulation, and dissemination of antibiotic resistance [18, 19] and indirectly influence the bacterial diversity and virulence in DFU [14, 20]. These findings suggest that the burden of antimicrobial resistance could be underestimated, particularly in the aspect of a growing population and climate change. Given this evolving and complex scenario of anti-microbial resistance, the need for novel eradication strategies to deal with polymicrobial infections in DFU is warranted.

Although the first-line therapies for DFU include surgical debridement, reducing pressure from weight bearing and infection management, the resistance towards antimicrobial therapy leads to chronically infected wounds that eventually lead to amputation. Hence, there is a requirement for novel strategies that can be applied in first-line treatments to counter DFU. In the current study, we characterize a custom-built, cold atmospheric plasma device and investigate the anti-bacterial efficacy of gram-negative and gram-positive bacteria in vitro and ex vivo.

## Materials and Methods

### Cold Atmospheric Plasma Device

**Fig. 1:**
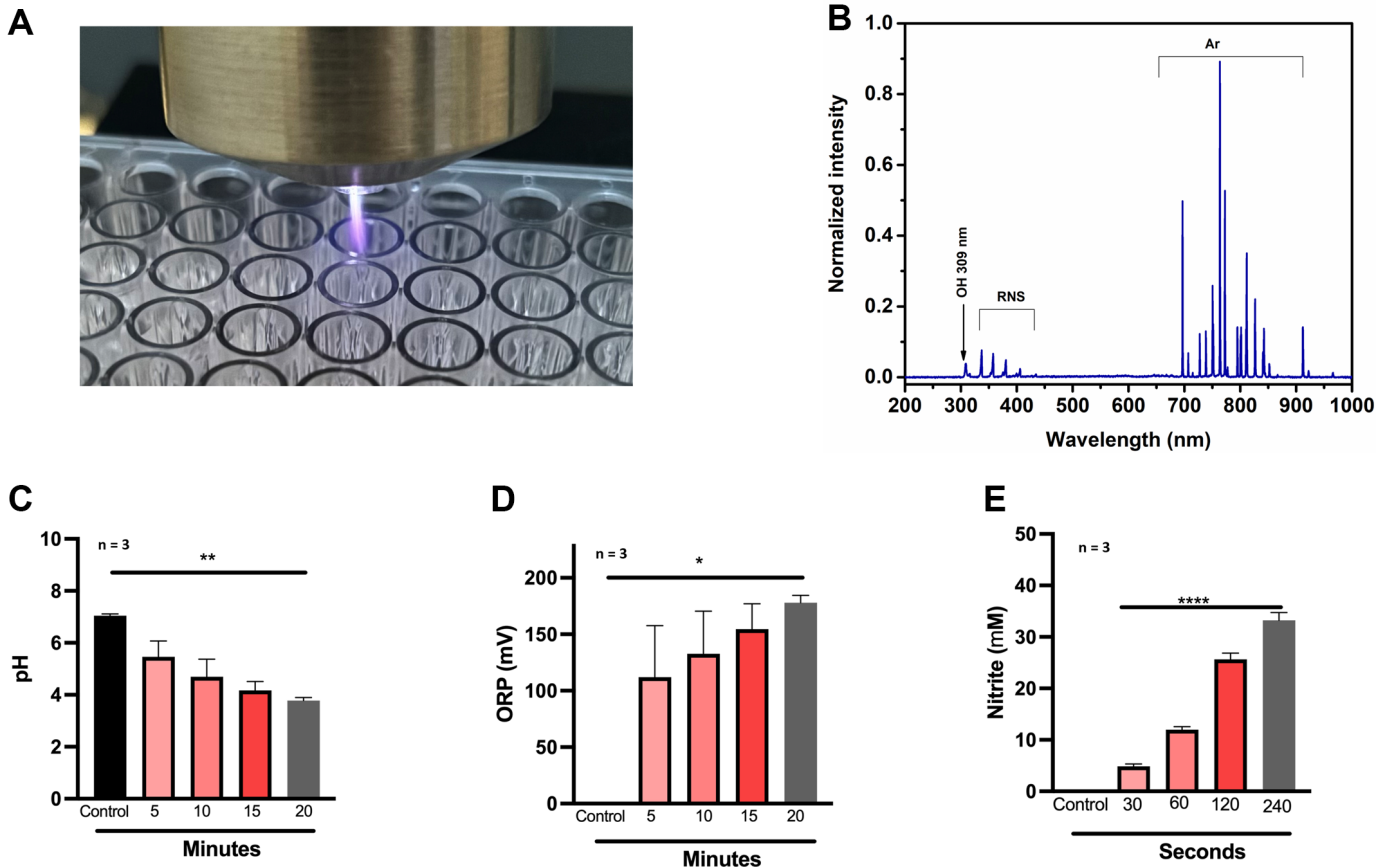
Development and characterization of cold atmospheric plasma jet system. (**A**) Cold Atmospheric Plasma (CAP) plume with argon feed gas; (**B**) Optical Emission Spectra of argon pulsed 9 kV plasma showing the presence of a significant amount of OH and N2 species; (**C**) Measurement of pH of distilled water treated with argon CAP for increased exposure times; (**D**) Measurement of ORP of distilled water treated with argon CAP for increased exposure times; (**E**) Quantification of nitrites using Griess assay with argon as feed gas for CAP. As a result, dose dependent accumulation of nitrite was observed. Data are mean ± SEM derived from three independent experiments with *p < 0.05, **p < 0.01, ***p < 0.001, ****p < 0.0001. Statistical analysis was done using ordinary one-way ANOVA.

A self-developed plasma reactor along with a high-frequency power supply was used in the present study. The device consists of the major components of a high-voltage power supply and plasma reactor. The plasma device consists of a glass tube with an inner diameter of 4 mm and a thickness of 1 mm which is enclosed in an aluminium casing. A photograph of a visible plasma jet formed in ambient air for a discharge voltage of 9 kV at the frequency of 48 kHz is shown in Fig. 1A. Experiments were performed with argon as a discharge gas with a flow rate of 2 slm. To analyze the discharge, the applied voltage and discharge current were respectively measured using a voltage probe (P6015A, Tektronix, 75 MHz bandwidth, USA) and a current probe (Tektronix P6021A Oscilloscope Probe 2 mA/mV). The electrical waveforms were recorded using a digital storage oscilloscope (Tektronix, TBS 1072 B, 70 MHz bandwidth, 1 GSa/s). The optical emission spectra were recorded using a spectrometer (HR-4XR, Ocean Optics, USA) to identify the reactive oxygen and nitrogen species (OH, NO, O, etc.). The collecting lens was placed at a 5 mm distance from the nozzle.

### Determination of Nitrites

A standard curve was constructed using serial dilutions of sodium nitrite (NaNO_2_) for the Griess assay. To determine the concentration of nitrites, PBS was treated with CAP for durations of 30 s, 60 s, 120 s, and 240 s. Non-ionized argon gas (120 s) was used as control. Equal volumes of plasma-treated PBS and Griess reagent were added to a 96-well flat-bottom plate. The plate was incubated for a period of 10 min in the dark and the absorbance was measured at 540 nm using a multi-mode plate reader (Molecular Devices^TM^, USA). The values of the unknown samples were then interpolated from the standard curve. Furthermore, pH and oxidation reduction potential (ORP) were measured in distilled water treated with CAP for 0, 10, 15 and 20 min using a multimode pH meter (Deep Vision^TM^, USA).

### Bacterial Strains

*Staphylococcus aureus*, *Enterococcus faecalis*, *Proteus mirabilis*, and *Pseudomonas aeruginosa* (MTCC strains) were obtained from the Department of Biotechnology, Sri Ramachandra Institute of Higher Education, (SRIHER) Chennai. Bacterial swabs from patients with diabetic foot ulcers were obtained from the Department of Plastic Surgery, Sri Ramachandra Medical College and Research Institute (SRMC&RI), SRIHER, Chennai, after prior informed consent. This study was approved by the Institutional Ethics Committee of SRIHER (CSP/23/DEC/140/931).

### Lawn Culture

*S. aureus*, *E. faecalis*, *Proteus mirabilis*, and *P. aeruginosa* were plated at 2 ×10^8^ CFU/ml, respectively, on LB agar plates and left to rest for 10min. Sinusoidal voltages of 9 kV at a frequency of 48kHz were applied to the CAP. Gas flow rates of 2 slm for argon were used. Individual plates were exposed to the plasma stream for 30, 60, 90 and 120s at a height of 10 mm. Non-ionized argon gas exposure for 120 s was used as negative control. Plates were incubated overnight at 37°C and then photographed using a digital transilluminator. Sterilization areas (%) were quantified using ImageJ, as previously described (21). Additionally, each image was spatially calibrated using the ratio of the area of inhibition to the area of the Petri dish to identify the percentage sterilization (%).

### Bacterial Isolates from Diabetic Foot Ulcers

Swabs from the deeper portion of the DFU were collected and directly inoculated into sterile culture tubes containing 5 ml of LB broth. The inoculated sample (500 μl) was used for culturing gram-negative and gram-positive bacteria by spread plate method using Eosin Methylene Blue (EMB) and mannitol agar, respectively. The following day, colonies were picked from the plates and starter culture (5 ml) was set in LB broth at 37°C overnight and a swab-full of this culture was used for plating on to pre-set agar plates and allowed to dry for 10 min. The plates were then exposed to CAP treatment as described earlier.

### Biofilm Assay

Microtiter biofilm assay was performed as previously described (22). The overnight polymicrobial starter culture was diluted 1:100 in fresh glucose broth. Then, 100 μl of the diluted bacteria broth was inoculated into a 96-well plate in replicates and incubated for 24 h at 37°C. After incubation, the medium was carefully decanted and washed once in phosphate-buffered saline (PBS). The wells with biofilm were then exposed to increasing durations of CAP (Control, 60 s and 120 s) and incubated for 15 min. The wells were then gently rinsed in 50 μl PBS and aliquoted to quantify free genomic DNA (A_260_) by spectrophotometer (Nanodrop 2000^TM^, USA). The wells were then treated with 100 μM resazurin and incubated for 2 h and fluorescence was measured using a multimode plate reader. The remaining replicates were rinsed once in PBS and stained with 0.1% crystal violet for 15 min. Wells were subsequently washed and imaged at 4X magnification in an inverted microscope (Nikon TS2^TM^, Japan) Alternatively, crystal violet was solubilized with 30% acetic acid in water for 15 min and the absorbance was measured at 550 nm using a multimode plate reader (Molecular Devices^TM^).

### Fast-Halo Assay

The peripheral blood mononuclear cells (PBMCs) were isolated from the heparinized blood of healthy volunteers (*n* = 3) by density gradient centrifugation (HiSep^TM^, USA). Then, 5 × 10^6^ cells in PBS were treated with CAP for durations of 60 s and 120 s and incubated for 1 h at 37°C. Cells were then transferred to RPMI 1640 media and cultured for another 24 h. Positive controls were included by treating the cells in similar conditions with 500 ng/ml lipopolysaccharide, and the feed gas-treated cells served as a negative control. The fast-halo assay was performed as previously described (23). Approximately 1.5 × 10^6^ cells were suspended in 40 μl of PBS, to which 140 μl of 0.75% low-melting agarose was added. About 60 μl of the agarose-cell suspension was layered onto the top of 1% normal agarose-precoated microscopic slides, and then covered with a coverslip. The slides were incubated at -20°C for 15 min to allow the gel to set. The coverslip was carefully removed. The non-denaturing lysis and staining of the fast-halo assay were performed on the prepared slides as described by Sestili *et al*. (2017). Upon treatment with lysis extraction buffer (50 mM NaOH, 100 mM NaH_2_PO_4_, 1 mM EDTA, and 1% Triton X-100 v/v, pH 11.6) for 5 min, the slides were washed with 1X PBS for 1 min and then treated with 8 μg/ml of PBS/RNase for 5 min. Finally, the slides were stained with Leishman Stain (HiMedia) for 10 min and washed with distilled water. The slides were air-dried prior to observation under light microscope. The images were captured using a Nikon ECLIPSE Ts2 microscope and analyzed using ImageJ software. The amount of DNA damage per cell is quantified in terms of nuclear diffusion factor (NDF), which is given by the formula:

NDF=[Halo area-Halo nuclei area/Halo nuclei area].

### Statistical Analysis

Graphing and statistical analysis were performed using GraphPad Prism 8.0. Difference between groups was analyzed using one-way ANOVA when comparing more than two groups with Tukey’s multiple comparison tests. *p* < 0.05 was considered statistically significant.

## Results

### Development and Characterization of the Cold Atmospheric Plasma Jet System

The CAP utilized in the current investigation was obtained from a self-designed device (Fig. 1A). The fiber-optic spectrometer was used to obtain the device’s optical emission spectrum (OES). The spectrum revealed emissions from different species, such as OH (309 nm), RNS (337-405 nm), and Ar (696.5-912 nm). This showed that the device produced both reactive oxygen and nitrogen species like N_2_, O^+^, N^+^ OH and NO (Fig. 1B). Moreover, to assess the stability of the device, the pH and millivolts were measured in deionized water exposed to CAP for 0, 5, 10, 15 and 20 min. The results indicated a dose-dependent decrease in pH (Fig. 1C). Previous studies indicated that CAP treatment alters the pH and oxidation-reduction potential (ORP). Similar observations were made in our study with CAP treatment increasing the ORP from 0 to a maximum of 150 mV after 20 min exposure (Fig. 1D). Nitric oxide (NO), also known as nitrogen monoxide, is a gaseous molecule considered one of the principal oxides of nitrogen. Production of NO results in the creation of an aqueous by-product, nitrite, which is used as an indirect measure of NO using the Griess assay. Deionized water was exposed to 30, 60, 120, and 180 s of CAP derived from argon and helium feed gases. Gas control (120 s) was used as negative control. We observed that argon-based CAP produced dose-dependent nitrites with a maximum of 34 μM after 120 s exposure (Fig. 1E). These results indicate that the custom-built CAP device consistently generates reactive species at both short-term and long-term exposures suitable for biomedical applications.

### Cold Atmospheric Plasma Inhibits Growth of Laboratory Gram-Positive and Gram-Negative Strains

**Fig. 2:**
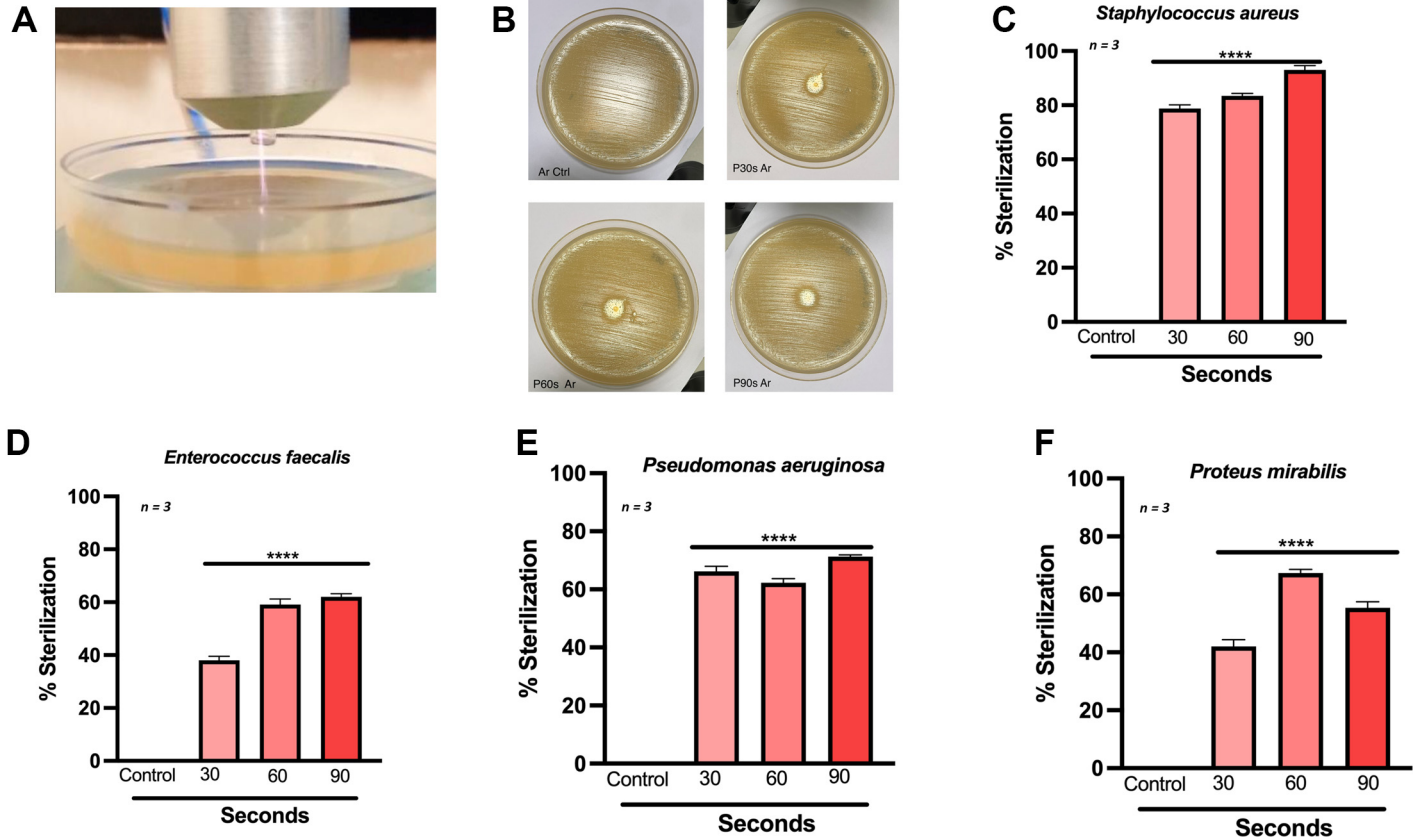
Cold Atmospheric Plasma inhibits growth of laboratory gram-positive and gram-negative strains. (**A**) Proximate image of treatment of bacterial agar plate using argon plasma jet; (**B**) Representative images of solid agar plate treated with argon CAP with varying exposure time; Sterilization of petri plates containing (**C**) *S. aureus* (**D**) *E. fecalis* (**E**) *P. Aeruginosa* and (**F**) *P. mirabilis* using argon CAP with varying exposure times. Data are mean ± SEM derived from three independent experiments with *p < 0.05, **p < 0.01, ***p < 0.001 and ****p < 0.0001. Statistical analysis was done using ordinary one-way ANOVA.

CAP generates a multitude of reactive oxygen and nitrogen species that have been demonstrated to target microbes. Both gram-positive and gram-negative bacteria have differential sensitivity towards oxidative stress. To determine the sterilization potential of the CAP device, we performed direct plasma treatment on gram-positive *S. aureus*, *E. faecalis* strains and gram-negative *P. aeruginosa* and *P. mirabilis* strains grown on lawn culture (Fig. 2A and 2B). These strains were first exposed to increasing duration of argon CAP for 30, 60, and 90 s, with non-ionized feed gas serving as control (90 s). These strains were exposed to increasing doses of CAP. The results indicate that *S. aureus* (Fig. 2C) and *E. faecalis* (Fig. 2D) had 80% and 60% sterilization after 60 s exposure, respectively. Similarly, *P. aeruginosa* (Fig. 2E) and *P. mirabilis* (Fig. 2F) had 60% sterilization after 60 s exposure. These results indicate that both gram-negative and gram-positive bacteria exhibited sensitivity to CAP treatment in vitro.

### Cold Atmospheric Plasma Inhibits Growth of Bacterial Strains Derived from DFU Patients

**Fig. 3:**
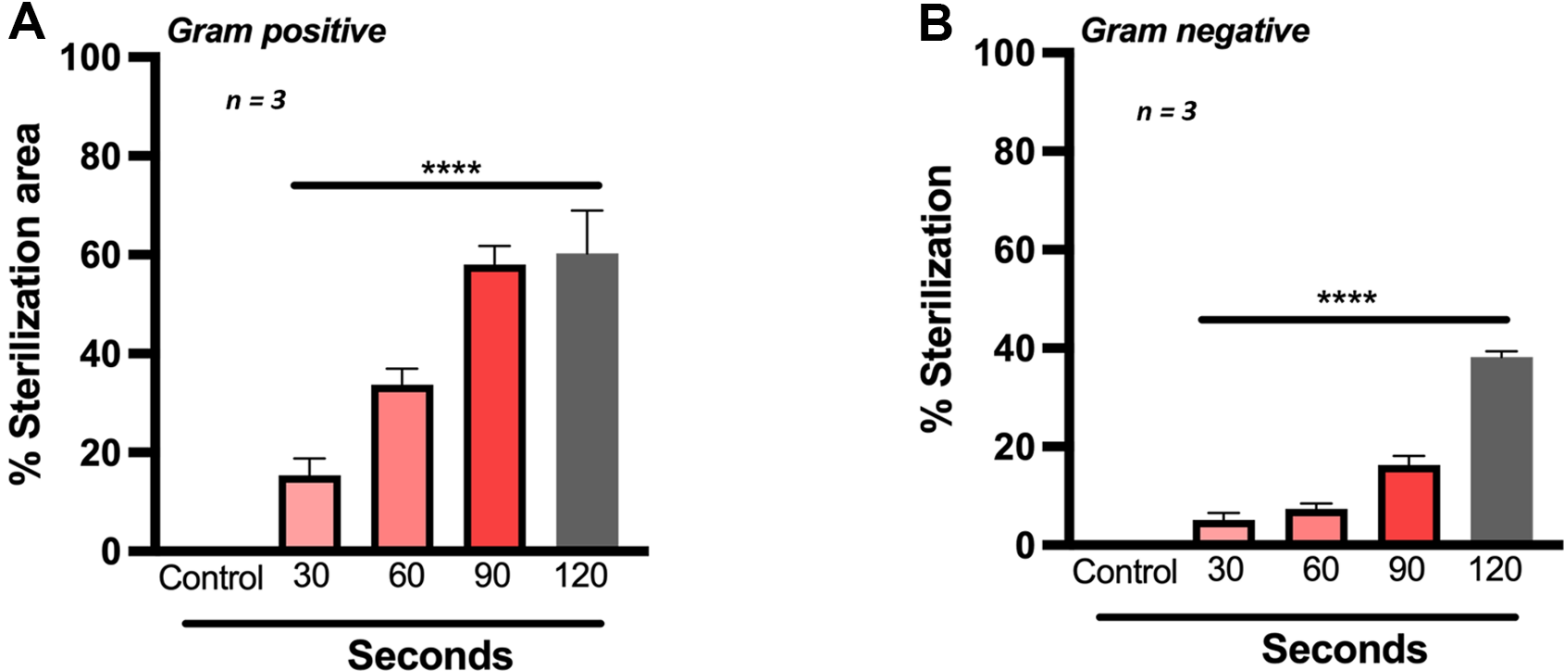
Cold Atmospheric Plasma inhibits growth of bacterial strains derived from Diabetic Foot Ulcer (**DFU**) patients. Sterilization of petri plates containing A. gram positive and B. gram negative bacteria isolated from patients with diabetic foot ulcers using argon CAP with varying exposure times. Data are mean ± SEM derived from three independent experiments with *p < 0.05, **p < 0.01, ***p < 0.001 and ****p < 0.0001. Statistical analysis was done using ordinary one-way ANOVA.

Bacterial swabs were obtained from patients with DFUs visiting the department of plastic surgery at SRIHER (Figs. S1, A-E). Patients were categorized based on their wound type, diabetic condition, and pre-treatment with antibiotics (Supplementary Table S1). Gram-positive and gram-negative bacterial strains derived from DFUs were maintained in selective agar media and exposed to increasing durations of CAP treatment. The results indicate a consistent and dose-dependent antibacterial effect of both gram-positive and gram-negative bacteria upon treatment with CAP (Figs. S2, A-B). In contrast to previous investigations, using CAP, gram-positive bacteria were more sensitive to CAP treatment when compared to gram-negative bacteria derived from DFUs. The increase in zone of inactivation was dose- dependent as seen at a maximum of 2 min exposure. The mean %sterilization area (*n* = 3) was around 60% in gram-positive and 39% with gram-negative bacteria, respectively (Fig. 3A and B).

### Cold Atmospheric Plasma Disrupts Growth of Biofilms Derived from DFU Patients

**Fig. 4:**
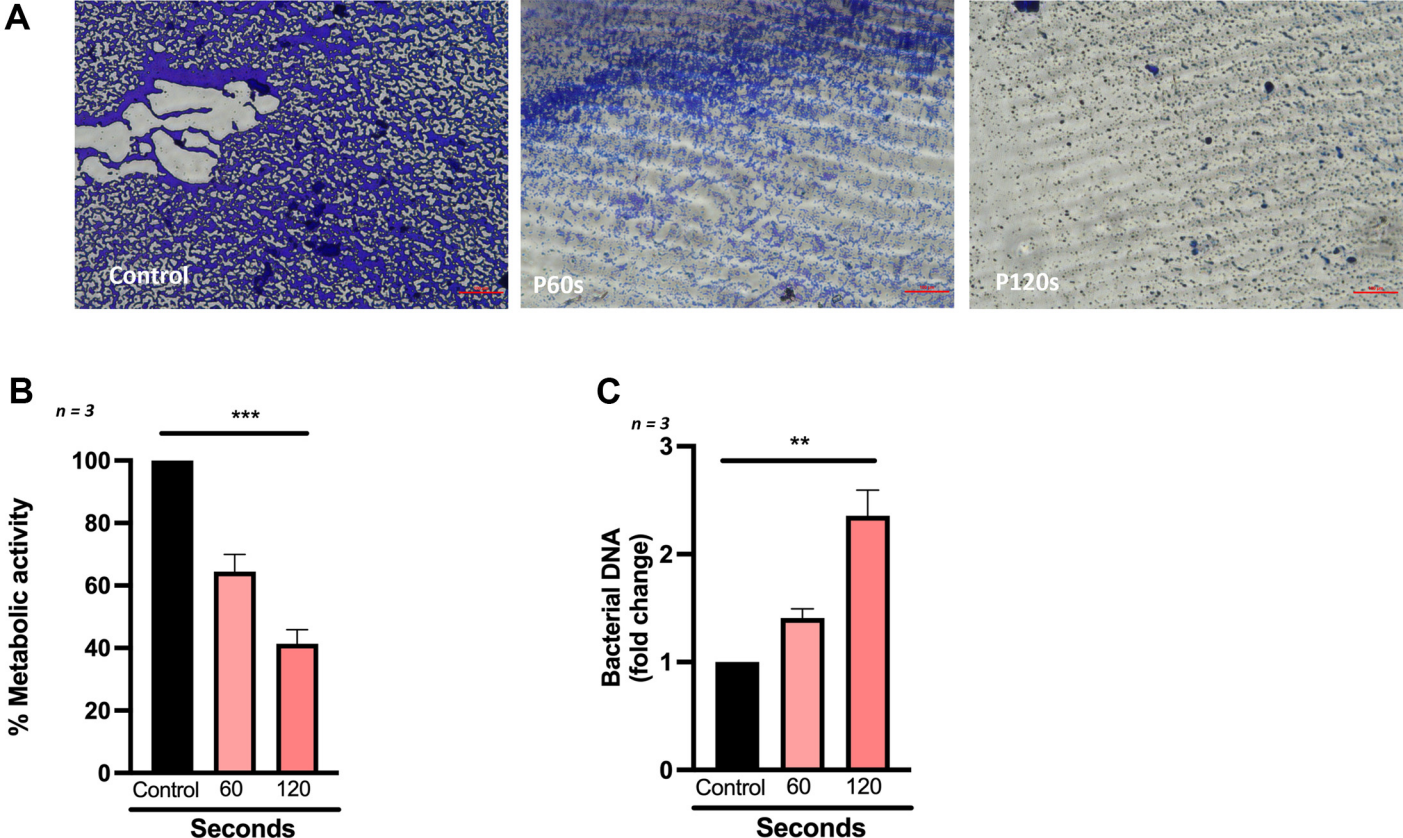
Cold Atmospheric Plasma disrupts growth of biofilms derived from diabetic foot ulcer (**DFU**) patients. (**A**) Representative images of polymicrobial biofilm treated with increasing exposure of CAP and stained with crystal violet; (**B**) Metabolic activity of bacteria present in biofilm post CAP exposure; (**C**) DNA (in fold change) released after lysis of bacterial cells after increased exposure of CAP. Data are mean ± SEM derived from three independent experiments with *p < 0.05, **p < 0.01, ***p < 0.001 and ****p < 0.0001. Statistical analysis was done using ordinary one-way ANOVA. Scale bar is 100 μm.

Biofilms are an integral part of DFUs, leading to resistance to treatment and enhanced virulence. Eradicating biofilms in chronic wounds is a perquisite for better treatment prognosis. To understand the efficacy of CAP on biofilms, in vitro assays were performed. CAP treatment significantly increased biofilm disruption in a dose-dependent manner as seen by crystal violet staining of 96-well plates treated with control, 60 s and 120 s of CAP exposure (Fig. 4A). The CAP exposure also led to a dose-dependent decrease of metabolic activity (alamarBlue) indicating eradication of biofilm (Fig. 4B). Furthermore, these wells were also quantified for free bacterial genomic DNA. There was a significant increase in fold change of free genomic DNA in treated conditions in comparison to controls, indicating the lysis of cells in the biofilm upon CAP treatment (Fig. 4C).

### Cold Atmospheric Plasma Exposure Does Not Induce Genotoxicity in Peripheral Blood Lymphocytes

**Fig. 5:**
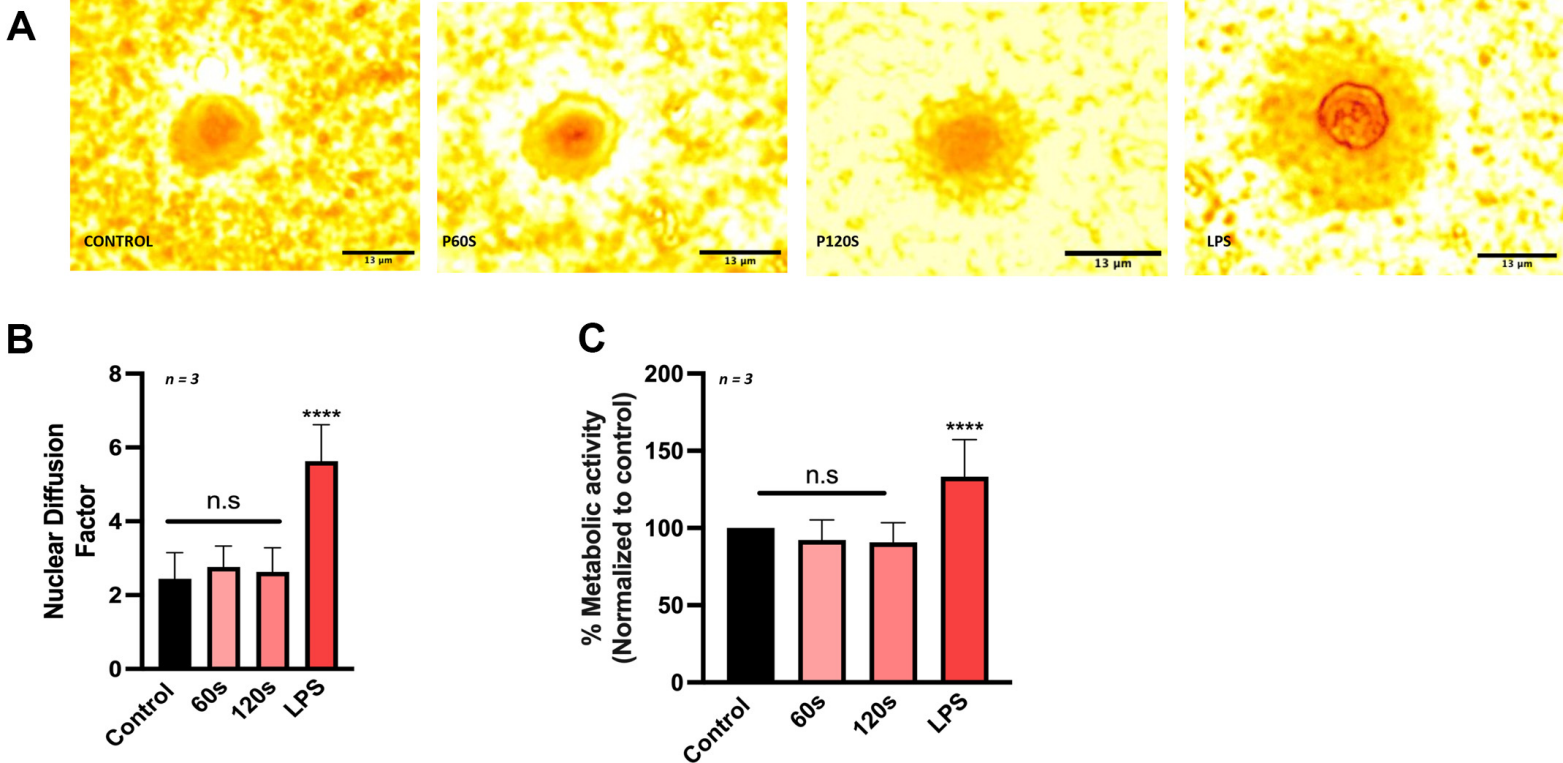
Cold Atmospheric Plasma exposure does not induce genotoxicity in Peripheral Blood Lymphocytes. (**A**) Representative images of fast halo assay performed on PBMCs treated with increasing exposure of CAP; (**B**) Nuclear diffusion in PBMCs treated with CAP and LPS (positive control); (**C**) Metabolic activity of PBMCs treated with CAP with different duration of exposures and with LPS. Data are mean ± SEM derived from three independent experiments with *p < 0.05, **p < 0.01, ***p < 0.001 and ****p < 0.0001. Statistical analysis was done using ordinary one-way ANOVA. Scale bar is 13 μm.

CAP is composed of several reactive oxygen and nitrogen species that have the potential to cause DNA damage at elevated levels. To understand the genotoxic potential of CAP, healthy peripheral blood lymphocytes were exposed to CAP at durations that led to elimination of bacteria. The results indicate there was no significant difference of nuclear damage seen from the fast-halo assay (Fig. 5A). The nuclear diffusion factor (NDF) was 2.66 ± 0.10 (control), 2.76 ± 0.11 (60 s), 2.67 ± 0.09 (120 s), and 5.95 ± 0.190 (LPS), respectively (Fig. 5B). Furthermore, there was no change in the metabolic activity after 24 h. These results indicate that CAP exposure effectively eliminates both gram-negative and gram-positive bacterial strains, sparing the normal lymphocytes from genotoxic insult.

## Discussion

Our CAP device is highly tuneable and capable of generating reactive oxygen and nitrogen species (RONS). Due to the differential redox status, the prokaryotic cells are eliminated by the RONS, whereas the eukaryotic cells are activated, leading to higher metabolic activity and proliferation upon CAP exposure [24]. This technology has several advantages over conventional therapies like hyperbaric oxygen therapy which involves breathing pure oxygen in a pressurized chamber that enhances oxygen delivery to tissues, thereby promoting wound healing [25]. However, such specialized equipment for hyperbaric oxygen therapy is not widely available, and furthermore, it is time-consuming and expensive to operate with several contraindications. Alternatively, CAP technology is a rapid, non-invasive and painless approach that is suitable for patients and can be easily integrated into routine wound care [24].

In this study, we characterized our custom-built CAP device and demonstrated its anti-bacterial activity in bacterial isolates derived from DFU. The device had consistent RONS production at lower as well as higher exposures. Gram-positive and gram-negative laboratory strains that are commonly found in DFU were sensitive to CAP exposure.*S. aureus*, which is the most prevalent pathogen in DFU and leads to abscesses, osteomyelitis, and gangrene [26], showed a robust inactivation of >70% in our study. *E. faecalis*, *P. aeruginosa*, and *P. mirabilis* had a > 60% inactivation in our study, indicating the potential for eliminating both gram-positive and gram-negative bacteria.

To validate the antimicrobial activity of CAP in microbial strains derived from patients with DFU, wound swabs were obtained during their clinical visit. Most of the patients had chronic DFU despite prior antibiotic pre-treatment. Ex vivo culture of wound swabs in selective agar differentiated gram-positive and gram-negative strains. CAP exposure led to a dose-dependent increase in % sterilization area in pre-lawned agar plates of gram-positive and gram-negative strains.

Interestingly, our study revealed gram-positive bacteria to be more sensitive to CAP than gram-negative microbes. Previous studies speculated that CAP-mediated bacterial inactivation is dependent on peptidoglycan thickness, differences in feed gas, and discharge configurations of the CAP, indicating that gram-positive bacteria are more resistant than gram-negative bacteria [27-29]. However, it has to be noted these studies were carried out in laboratory strains under a controlled environment (21). In our study, we observed that gram-negative bacteria were less sensitive to CAP treatment in comparison to gram-positive bacteria. Our studies were carried out using swabs from DFU infected with polymicrobial strains. It has been previously reported that bacteria possess a robust mechanism to evade chemical-based therapies by means of a thick outer membrane, efficient efflux pumps, and altered porin channels. CAP is a non-chemical therapeutic strategy (directly deposits RONS), and the major factors that play a key role in the reduced sensitivity in gram-negative bacteria are higher glutathione content, high lipid content, presence of periplasmic superoxide dismutase (SOD), and enhanced DNA repair mechanisms [30, 31].

The wounds presented in the clinic are of larger dimensions due to chronic diabetes and lack of awareness in the rural population. Treating larger wound areas with CAP would be a time- consuming process in a healthcare setting. This warrants customized CAP devices designed for treatment of large diabetic foot ulcers, as well as the integration of this modality in existing standard care therapies.

Finally, to mimic the clinical setting, poly-bacterial biofilm inactivation assays were performed from DFU swabs. CAP significantly disrupted the biofilm load following 60 and 120 s exposure. The non-ionized argon feed gas exposure (120 s) did not disrupt the biofilm activity in our study.Previous studies suggested that a 2 h incubation period is optimal to assess the bacterial inactivation following CAP treatment [21]. Other studies previously characterized the biofilm eradication in mono-bacterial laboratory strains [27, 32, 33]. Our study demonstrated the efficacy of CAP treatment in poly-bacterial biofilm derived from DFU within the first 15 min ex vivo. The reduced metabolic activity also led to finding increased free bacterial DNA in the supernatant post-CAP treatment. Pathogens-associated molecular patterns (PAMPs) are microbial structures and DNA which are crucial for survival and virulence and are recognized by evolutionarily conserved pattern recognition receptors (PRRs) in eukaryotic cells, thereby activating innate immune responses. Toll-like receptors (TLRs) play a crucial role in eliciting early mechanisms of host defenses but also prime antigen-specific adaptive immune responses [34]. Endosomal TLR3 and TLR9 recognize double-stranded DNA and CpG bacterial DNA, respectively [35]. Rapid release of PAMPs from biofilms following CAP exposure has the potential to evoke innate immune response to synergize the elimination of infections in DFU. Several studies have previously documented the safety of CAP exposure in human cells and tissues [36]. In our study, we used primary PBLs and identified that CAP exposure has no significant effect on DNA damage or change in the metabolic state ex vivo. This is due to the robust DNA damage response in healthy cells that have an inherent capacity to repair double-strand and single-strand breaks effectively [37].

Collectively, our data report the first clinical relevance of CAP in eliminating bacterial pathogens in DFU ex vivo in the Southern Indian population. Although this technology has seen success in several randomized clinical trials [38-40], satisfying primary (wound disinfection) and secondary objectives (rapid wound closure), along with further investigations are necessary to identify the optimal CAP parameters against the microbial diversity in the Indian population, as well as the clinical protocols, while also reducing antibiotic dependency and enhancing wound healing.

## Supplemental Materials

Supplementary data for this paper are available on-line only at http://jmb.or.kr.
