# Supplementary material for: Antimicrobial Activity of Cold Atmospheric Plasma on Bacterial Strains Derived from Patients with Diabetic Foot Ulcers
Source: J Microbiol Biotechnol. 2024 Sep 30;34(11):2353–61. doi: 10.4014/jmb.2407.07035 (PMC11637820; doi:10.4014/jmb.2407.07035)
Supplement: Supplementary file 1 [file jmb-34-11-2353-supple.pdf]

J. Microbiol. Biotechnol. 2024. 34(11): 2353–2361
https://doi.org/10.4014/jmb.2407.07035

Antimicrobial Activity of Cold Atmospheric Plasma on Bacterial Strains Derived from Patients with Diabetic Foot Ulcers

Supplementary Table S1. Details of diabetic foot ulcer patients participated in the study.

SAMPLE ID	AGE	SEX	DIABETES TYPE	WOUND TYPE	ANTIBIOTIC PRE-TREATMENT	CULTURE OBTAINED	
						GRAM POSITIVE	GRAM NEGATIVE
DFUGA_01	56	Male	Type II Diabetes	Wet ulcer	Multiple antibiotics	✓	✓
DFUMU_02	41	Male	Type II Diabetes	Wet ulcer	Livofloxacin	✓	
DFUTA_03	62	Male	Type II Diabetes	Wet ulcer	Linezolid	✓	✓
DFUPA_04	58	Male	Type II Diabetes	Wet ulcer	Faronem		✓
DFUSU_05	61	Male	Type II Diabetes	Wet gangrene	No pre-treatment	✓	✓

Supplementary figure 1

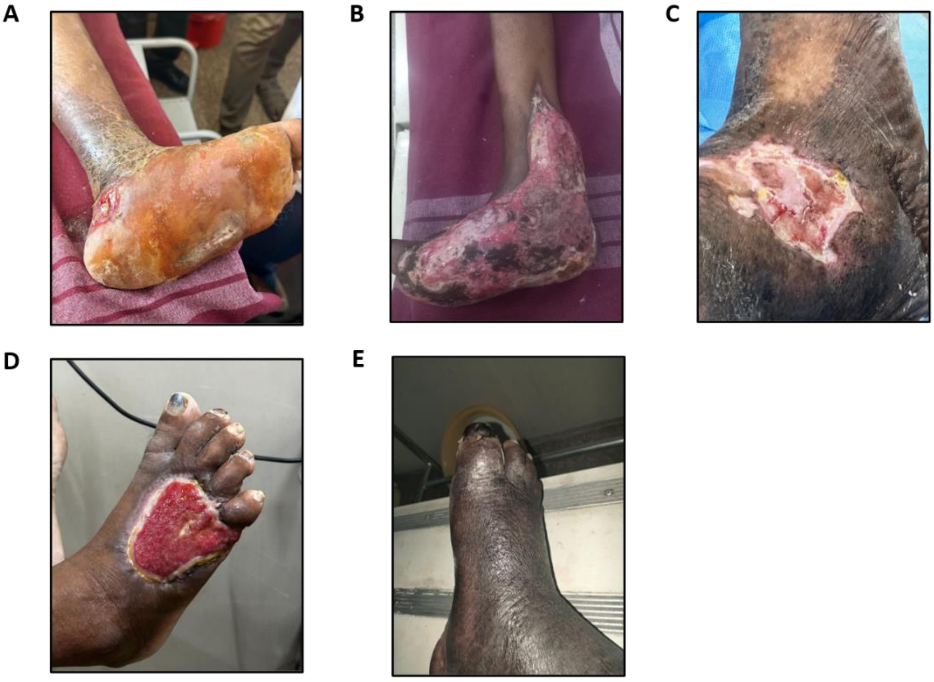

Fig S1. Representative images of diabetic foot ulcers of patients **A.** DFUGA_01 **B.** DFUMU_02 **C.** DFUTA_03 **D.** DFUPA_04 and **E.** DFUSU_05

Supplementary figure 2

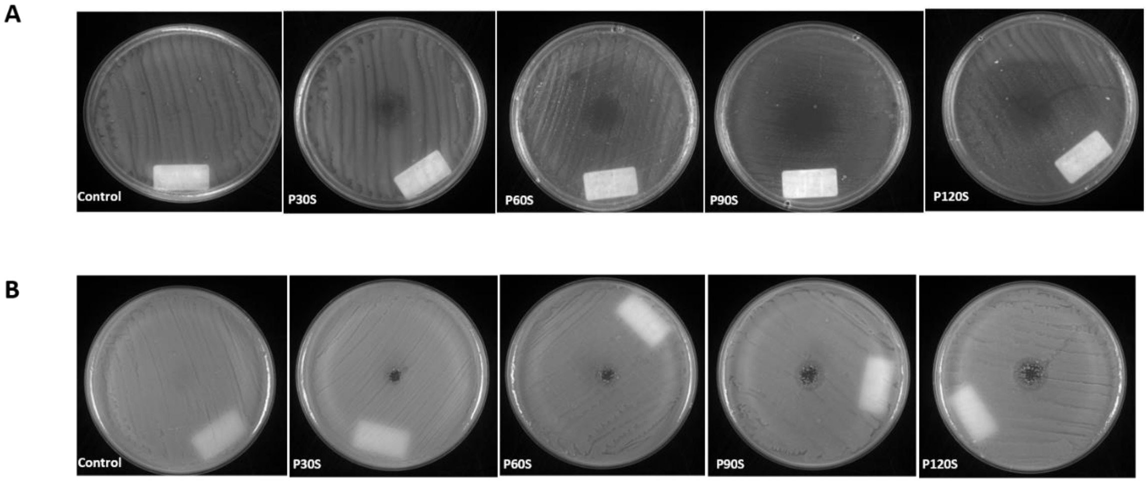

Fig S2. Representative images of agar plates containing **A.** gram positive bacteria and **B.** gram negative bacteria isolated from diabetic foot ulcers and treated with argon CAP with increased exposure times that shows zone of inactivation.

Supplementary figure 3

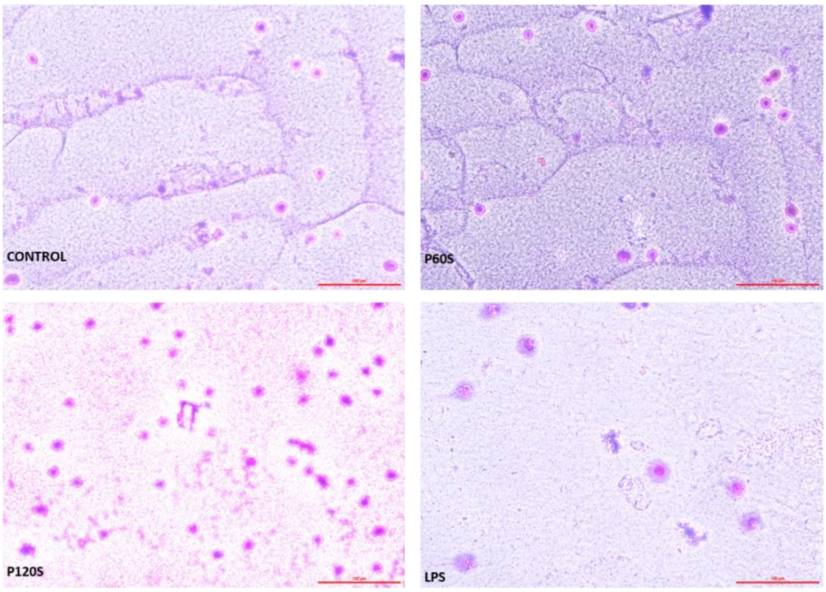

Fig S3. Representative images of fast halo assay performed in PBMCs treated with increasing exposure of CAP and with LPS. Scale bar is 100 µm.
